# Association between acetylcholinesterase inhibitors and risk of stroke in patients with dementia

**DOI:** 10.1038/srep29266

**Published:** 2016-07-05

**Authors:** Yi-Ting Lin, Ping-Hsun Wu, Cheng-Sheng Chen, Yi-Hsin Yang, Yuan-Han Yang

**Affiliations:** 1 Graduate Institute of Clinical Medicine, College of Medicine, Kaohsiung Medical University, Kaohsiung, Taiwan; 2 Department of Family Medicine, Kaohsiung Municipal Hsiao–Kang Hospital, Kaohsiung, Taiwan; 3 Department of Family Medicine, Kaohsiung Medical University Hospital, Kaohsiung, Taiwan; 4 Division of Nephrology, Department of Internal Medicine, Kaohsiung Medical University Hospital, Kaohsiung, Taiwan; 5 Department of Psychiatry, Kaohsiung Medical University, Kaohsiung, Taiwan; 6 Department of Psychiatry, Kaohsiung Medical University Hospital, Kaohsiung, Taiwan; 7 School of Pharmacy, College of Pharmacy, Kaohsiung Medical University, Kaohsiung, Taiwan; 8 Department of Neurology, Kaohsiung Medical University, Kaohsiung, Taiwan; 9 Department of Neurology, Kaohsiung Medical University Hospital, Kaohsiung, Taiwan; 10 Department of Neurology, Kaohsiung Municipal Ta-Tung Hospital, Kaohsiung, Taiwan

## Abstract

Patients with dementia are at increased risk of stroke. Acetylcholinesterase inhibitors (AChEIs) have endothelial function protection effects and anti-inflammatory properties. We investigated the ischemic stroke risk in AChEIs use in dementia patients without stroke history. Using Taiwan National Health Insurance Database from 1999 to 2008, 37,352 dementia patients over 50 years old without stroke history were eligible. The results were analyzed by propensity score–matched Cox proportional hazard models with competing risk adjustment. AChEIs users had lower incidence of ischemic stroke (160.3/10,000 person-years), compared to the propensity score–matched reference (240.8/10,000 person-years). The adjusted hazard ratio for ischemic stroke based on propensity score–matched Cox proportional hazard model was 0.508 (95% confidence interval, 0.434–0.594; P < 0.001). There was no significant difference in all-cause mortality between AChEIs users and nonusers. In conclusion, among dementia patients without previous ischemic stroke history, AChEIs treatment was associated with a decreased risk of ischemic stroke but not greater survival.

According to recent estimates, there are 24.3 million cases of dementia globally, with that number expected to reach 81.1 million cases in 2040^1^. Increasing epidemiologic evidence shows that dementia itself is associated with an increased risk of ischemic stroke^2^,^3^,^4^. Dementia patients with concurrent stroke attack have accelerated functional decline, decreased daily activities, and reduced survival^5^,^6^,^7^. These patients also tend to have poor quality of life, and their care places a greater economic burden on themselves, their families, and society^8^,^9^.

Acetylcholinesterase inhibitors (AChEIs), which have beneficial effects on cognition function^10^, are currently approved for the treatment of Alzheimer’s disease (AD) and licensed for the treatment of vascular dementia, with several clinical benefits^11^,^12^. Several experimental studies suggested that AChEIs also have anti-inflammatory properties^13^,^14^,^15^ and protect endothelial cells^16^,^17^. Because endothelial cells play an important role in ischemic stroke development, we assumed that AChEIs may benefit endothelial cell function and reduce atherosclerosis by blocking the inflammatory process, further lower the incidence of cerebrovascular diseases such as cerebral ischemic infarction.

Thus, we conducted a retrospective analysis based on the Taiwan National Health Insurance Research Database (NHIRD), to investigate whether AChEIs use is associated with a lower risk of ischemic stroke among patients with dementia.

## Methods

### Database

This population-based cohort study utilizes the Taiwan NHIRD, which has been prospectively collecting nationwide health care data since the Taiwan National Health Insurance (NHI) was implemented in 1995^18^. The database consists of detailed health care data for over 23.7 million enrollees, representing more than 99% of Taiwan’s entire population, and it includes complete outpatient visits, hospital admissions, prescriptions, disease, and vital status. The NHIRD also includes a registry system for “catastrophic illnesses”, including dementia, cancer, end-stage renal disease, and several autoimmune diseases. The database contains all relevant information about the catastrophic illness status, including diagnostic codes based on the *International Classification of Disease, Ninth Revision* (ICD-9), dates of diagnosis, dates of death, dates of clinic visit, details of prescriptions, expenditure amounts, and outpatient/inpatient claims data. Because each individual registered in the catastrophic illnesses database is exempted from any co-payment for treatment, the registry is comprehensive. The Institutional Review Board of Kaohsiung Medical University Hospital approved this study (KMUH-IRB-EXEMPT-20130062).

### Study population and cohort

From the Catastrophic Illness Patient Registry, we selected all patients diagnosed with dementia, defined as those who had catastrophic illness registration for dementia (ICD-9 code 290, 331.0) between January 1, 1999, and December 31, 2008. We excluded individuals younger than 50 years (n = 689) and those who had been hospitalized for ischemic stroke (n = 2112). Of a total of 42,594 patients with dementia and no ischemic stroke hospitalization history, we generated a propensity score-matched cohort of 10,364 patients (5182 exposed and 5182 unexposed to AChEIs) for our outcomes analysis (Supplementary Figure).

### Covariates and propensity score matching

Baseline demographic data and information on clinical conditions were obtained for all individuals in both cohorts from inpatient and outpatient reimbursement data in NHIRD. We identified the following comorbidities as potential confounders: diabetes mellitus, hypertension, hyperlipidemia, coronary artery disease, heart failure, atrial fibrillation, peripheral artery disease, cerebrovascular disease, chronic obstructive pulmonary disease, chronic kidney disease, malignancy, and depression (Supplementary Table S1). Socio-demographic characteristics (age, sex, income, and the level of urbanization) were also taken into consideration in our analysis. Urbanization levels in Taiwan are divided into three strata according to the Taiwan National Health Research Institute publications. The income served as a proxy indicator of economic status, which was classified as one of three categories: fixed premium and dependent, less than New Taiwan Dollars (NTD)20,000 monthly, or NTD20,000 or more monthly (US$1 = NTD32.1 in 2008).

Using a logistic regression model, we determined a propensity score for AChEIs users within the exposure period. The covariates entered into the propensity score were age, sex, socio-demographic characteristics (living arrangements and economic status), and comorbidities (Table 1).

### Exposure to AChEIs and use of other drugs

Dementia patients received prescriptions of AChEIs (N06DA02, N06DA03, and N06DA04 according to the Anatomical Therapeutic Chemical classification system). In Taiwan, claims for AChEIs prescriptions in patients with dementia must undergo a special review process to assess the patient’s detailed medical records, biochemistry data (including complete blood cell count, venereal disease laboratory results, blood urea nitrogen, creatinine, alanine aminotransferase, aspartate aminotransferase, thyroxine, and thyrotropin), and neuroimages (at least one report of computed tomography, magnetic resonance imaging, or Hachinski ischemic score). The review is conducted by a Bureau of NHI committee consisting of neurologists or psychiatrists^4^. The defined daily dose (DDD) recommended by the World Health Organization is a unit for measuring a prescribed amount of drug; specifically, it is the assumed average maintenance dose per day of a drug consumed for its main indication in adults. By using the following formula, we could compare any AChEIs based on the same standard: (total amount of drug)/(amount of drug in a DDD) = number of DDDs^19^. Cumulative DDDs (cDDDs), the sum of dispensed DDDs of any AChEI, served as the duration of AChEI exposure to compare the drugs’ use to the risk of ischemic stroke. To examine the dose–response relationship, we defined three dosage groups in each cohort: <28, 28 to 365, and >365 cDDDs. Patients who used AChEIs for less than 28 cDDDs were considered AChEIs nonusers in the dose–response relationships models.

We also retrieved details on other medications used during the cohort observation period, including antiplatelets, dipyridamole, warfarin, angiotensin-converting enzyme inhibitors, angiotensin receptor blockers, beta-blockers, thiazides, calcium channel blockers, statins, fibrates, traditional nonsteroidal anti-inflammatory drugs, cyclooxygenase-2–selective inhibitors, proton pump inhibitors, histamine-2 receptor antagonists, antidepressants, and antipsychotics (Supplementary Table S2).

### Measurement of outcomes

Our primary outcome was the occurrence of ischemic stroke, which was defined as admission to a hospital for acute ischemic stroke. Secondary outcomes included all-cause death during the study period. The diagnoses of ischemic stroke have been validated in the national patient registry^20^. The study end points were followed until primary outcome, death, or 2009.

### Patients with AD subgroup analysis cohort

In order to confirm results, we further analysis patients with AD using the same study protocols. In AD subgroup cohort, we selected 5872 patients diagnosed with AD (ICD-9 code 331.0) and no ischemic stroke hospitalization history. We identified 2649 patients who were treated by AChEIs and matched each of these patients with an untreated control according to age, sex, and index date of AChEIs prescription. These patients were also followed until primary outcome, death, or 2009.

### Statistical Analysis

Baseline descriptive data are described as mean ± standard deviation for continuous variables and frequency and percentage for categorical variables.

Because AChEIs users and nonusers had different baseline characteristics, we quantified a propensity score to compare risk of stroke in groups with and without AChEIs treatment. We used one-to-one matching without replacement, with a caliper width of 0.2 of the standard deviation of the logit of the propensity score using multivariate logistic regression analysis, conditional on the baseline covariates specified in Table 1. Then, we carried out a propensity score–matched Cox proportional hazard analysis that was conditional on competing risk of death (Fine and Gray competing-risk models^21^) to derive hazard ratios and 95% confidence intervals in relation to the primary outcomes. The proportional hazard assumption of the Cox models was assessed by a graphical method. To assess the robustness of our results, we performed a series of additional analyses, including a dose- response analysis and stratified analysis according to baseline characteristics. Because the recurrence of ischemic stroke in AChEIs treatment and comparison groups has the competing risk for death, we used the modified Kaplan-Meier method^22^ to estimate the cumulative incidence rates of ischemic stroke for the two groups. In order to check the confounding effect of the newly developed cerebrovascular disease during the follow-up period, the exposure to AChEIs was treated as a time-dependent variable. Time-dependent Cox regression was performed to estimate the hazard ratios for ischemic stroke among AChEI users versus non-users and for the tertiles of the cDDDs to avoid time-varying prescription changes. Furthermore, multivariable analyses were used to evaluate linear trends in risk by treating AChEI use as a continuous variable after assigning a score to each exposure level. The *P*-value was calculated for trends to confirm the dose-response relationship. All analyses were performed using the SAS statistical software (version 9.2). Calculations of cumulative incidence in the competing risk analysis were carried out using the “cmprsk” package R (http://cran.r-project.org/web/packages/cmprsk/index.html). All statistical tests were two sided. A *P*-value < 0.05 was considered statistically significant.

## Results

### Baseline characteristics

To estimate the probability of receiving AChEIs therapy, age, sex, socio-demographic characteristics (living arrangements and economic status), and comorbidities (in separate terms for individual comorbidities) were used to calculate propensity scores. Finally, we recruited 5182 patients into the treated cohort and 5182 patients into the untreated cohort. The characteristics of the two groups were balanced after matching by the propensity score (Table 1). The mean follow-up durations for the treated and untreated cohorts were 5.03 and 5.04 years, respectively (Table 2).

### Outcomes of ischemic stroke and all cause of mortality

**Figure 1:**
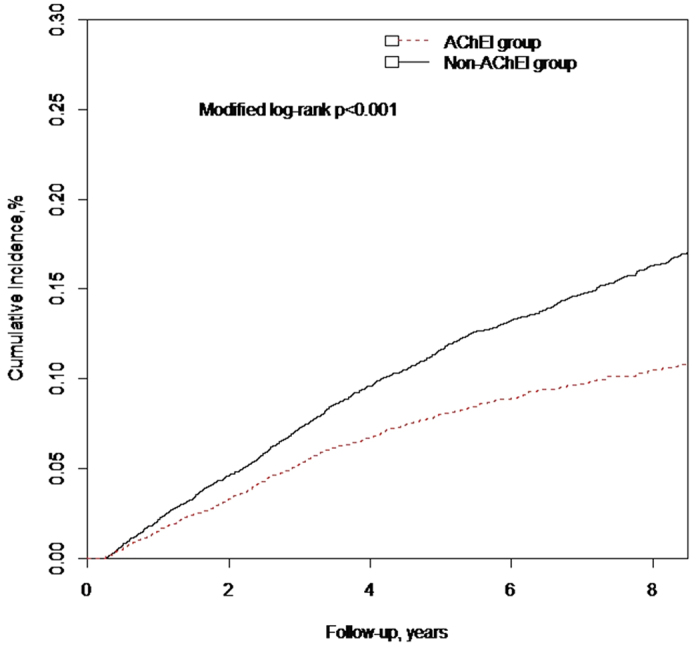
Cumulative incidences of ischemic stroke of dementia patients who were or were not treated with acetylcholinesterase inhibitors. Data were compiled after adjustment for competing mortality. For cumulative incidences of ischemic stroke, calculation and comparison in competing risk data ratios were conducted using modified Kaplan-Meier and Gray methods.

AChEIs users had a lower incidence rate of ischemic stroke than nonusers (incidence rate of 160.3 [95% CI, 145.5–176.2] per 100,000 person-years vs 240.8 [95% CI, 222.5–260.2] per 100,000 person-years) (Table 2). The HRs in propensity score–matched modified Cox proportional hazards models were 0.655 (95% CI 0.579–0.742; *P* < 0.001) and 0.508 (95% CI 0.434–0.594; *P* < 0.001) after further concomitant medications adjustment (Table 3). There were no differences in the effects of different AChEIs on stroke (data not shown). The cumulative incidences of ischemic stroke were lower in AChEIs users than in their matched controls (*P* < 0.001) based on analysis using the modified Kaplan-Meier approach with adjustment for competing risk of mortality (Fig. 1). We observed a dose–response relationship between AChEIs use and ischemic stroke risk. The propensity score–matched time-dependent modified Cox proportional analysis HRs were 0.646 (95% CI 0.567–0.736; *P* < 0.001) and 0.587 (95% CI 0.512–0.672; *P* < 0.001) for AChEIs cDDDs of 28–365 and >365, respectively, relative to no AChEIs use (<28 cDDDs) There was a significant trend toward risk reduction with increasing doses of AChEIs (P for trend < 0.001) (Table 4).

As for death, AChEIs users were nonsignificant associated with a lower risk of all-cause mortality compared with nonusers in propensity score–matched Cox proportional hazards models (HR, 0.947, 95% CI, 0.875–1.024; *P* = 0.063 ; Model 1) and still nonsignificant after further adjusting for concomitant medications (adjusted HR, 0.996, 95% CI, 0.872–1.137; *P* = 0.193; Model 2) (Table 3).

### Stratified analyses for ischemic stroke separately

**Figure 2:**
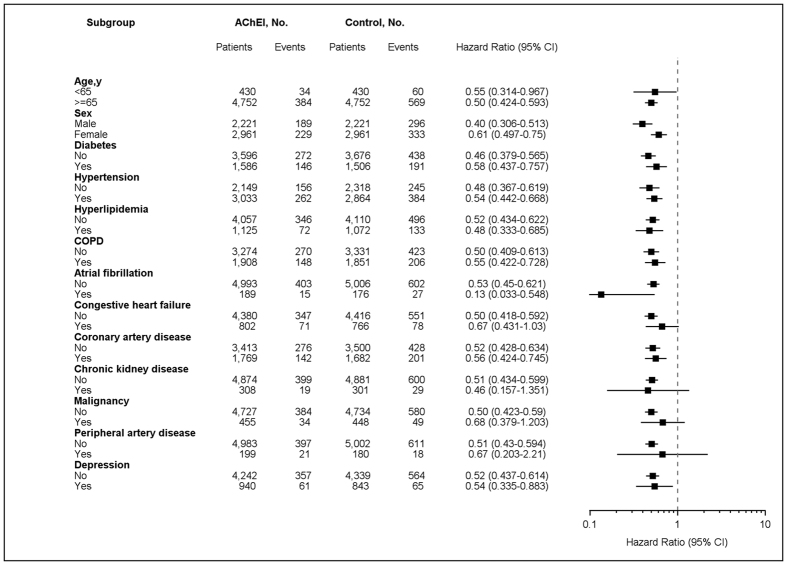
Stratified analysis for ischemic stroke in propensity score–matched cohorts. The risk of ischemic stroke in dementia patients with and without use of acetylcholinesterase inhibitors (presented by hazard ratios and 95% confidence intervals) is shown, stratified by the baseline characteristics.

Analyses of propensity score–matched subgroups of dementia patients with AChEIs treatment are shown in Fig. 2. The association with a lower risk of ischemic stroke was observed in most strata except in patients with heart failure, chronic kidney disease, malignancy, and peripheral artery disease.

### Outcomes of patients with AD subgroup cohort

In patients with AD cohort, the incidence rate of ischemic stroke was 166.4 (95% CI, 145.8–189.1) per 100,000 person-years in AChEIs users and 193.1 (95% CI, 171.2–217.7) per 100,000 person-years in nonusers (Supplementary Table S4). The cumulative incidences of ischemic stroke were lower in AChEIs users than nonusers (Log rank *P* < 0.001) (Supplementary Figure 2). In the modified Cox regression analysis adjusting for urbanization, socioeconomic status, and comorbidities, AChEIs users showed a lower risk of ischemic stroke (adjusted HR, 0.626, 95% CI, 0.512–0.767; *P* < 0.001; Model 2) than nonusers. The lower risk of ischemic stroke was still significant after additionally adjusting for concomitant medications (adjusted HR, 0.615, 95% CI, 0.498–0.760; *P* < 0.001; Model 3) (Supplementary Table S5). A dose–response relationship between AChEIs use and ischemic stroke risk was still evident in the patients with AD. The adjusted HRs were 0.779 (95% CI, 0.610–0.994; *P* = 0.045) and 0.585 (95% CI, 0.456–0.750; *P* < 0.001) for patients with cDDDs of 28 to 365 and >365 compared to those with cDDDs <28. There was a trend toward risk reduction with increasing cDDD of AChEIs in the patients with AD (*P* < 0.001) (Supplementary Table S6).

## Discussion

In this observational retrospective cohort study of patients with moderate to severe dementia, AChEIs treatment was associated with a decreased risk of ischemic stroke events. To clarify possible dose–response relationships, we proposed the concept of cDDDs and detected a statistically significant inverse trend between the dose and the cumulative incidences and HRs of ischemic stroke in dementia patients. Further sensitivity analysis focused on the AD cohort showed that the results were robust. Our results suggest that using AChEIs not only treats cognitive function declines but also ameliorates ischemic stroke risk.

There are some potential mechanisms by which AChEIs could decrease risk of ischemic stroke. First, dementia might exacerbate atherosclerosis and cerebral vascular dysfunction^4^. AChEIs may have an adjunctive protective role on endothelial dysfunction related to dementia^23^ based on antiapoptotic effects on endothelial cells and mechanisms against oxidative stress–induced cytotoxicity^16^. Second, atherosclerosis is considered to be an inflammatory disease^24^. The anti-inflammatory effect of AChEIs due to reduced acetylcholine breakdown is of interest^25^,^26^,^27^. Treatment with AChEIs is associated with reduction in the serum cytokine level and cytokine production^14^,^15^ that would, in part, have a reduction in ischemic stroke events as observed in our study.

Similar findings have been reported in a Swedish study, which found that AChEIs use was associated with 38% lower risk of myocardial infarction in patients with AD, 36% decreased risk of death, and 26% reduced risk of death from cardiovascular causes^28^. Our study did not reveal a significant association between death and use of AChEIs, which was partly different from the Swedish study, which excluded patients with high risks of cardiovascular disease. Although use of AChEIs might lower mortality risk, studies addressing the relationship between AChEIs use and mortality are conflicting^29^,^30^. Furthermore, differences also existed in study samples, baseline characteristics, early or late start in AChEIs therapy, and study methodologies. In addition, the mortality rates of patients with dementia in Taiwan, which population-based studies report as ranging from 32% to 48%^31^, are higher than those reported in Western populations^32^. The different mortality rates based on country and race might explain the results regarding AChEIs effects on death risk in the present study.

Our study has several strengths. The ascertainment of ischemic stroke hospitalization and medical comorbidities are complete, objective, and reliable because NHI is a compulsory and universal health care system with a very high coverage rate in Taiwan. Administrative databases allowed for the follow-up of each patient, allowing us to avoid attrition over time and minimizing the possibility of recall bias. In addition, virtually all hospitalizations for ischemic stroke used essential diagnostic procedures, including brain computed tomography or magnetic resonance imaging, which further increased the validity of stroke diagnoses. Finally, we used multiple strategies to minimize confounding. Propensity score helped to mitigate bias due to confounding by indication, as well as balance a wide range of cardiovascular risk factors between groups. Although unmeasured confounders may still exist, we believe the methodology used in the present study is solid and robust. Nevertheless, several limitations need to be considered. First, the study was retrospective, based on review of the medical records in a database and not on formal cognitive function testing. Dementia coding has been reported to have low sensitivity but high specificity^33^. Ascertainment of dementia based on ICD-9 coding was accurate in the present study because it was well validated in the catastrophic illness registry. However, dementia severity was not available in NHIRD. Second, the diagnosis of various comorbidities was based on claims data and ICD-9 code, which may be associated with potential misclassification bias. Third, the NHIRD lacks data on individual behaviors and information. Thus, other potential confounding factors such as smoking, physical inactivity, family history, and laboratory data were not available. Fourth, the present study included only mild-to-moderate dementia cases with the treatments of AChEIs. Patients with severe dementia or without receiving treatments with AChEIs were therefore omitted from analyses. Therefore, the results might not be generalized to all dementia populations. Finally, the study included Taiwanese patients only. Whether the findings are also applicable to other ethnic population requires further evaluation.

In conclusion, this observational propensity score matched cohort study indicated that use of AChEIs in dementia patients without ischemic stroke hospitalization history was associated with a decreased risk of an ischemic stroke event but not all-cause mortality. Focus on this area, randomized prospective studies, and observational studies based on other populations are needed.

## Additional Information

**How to cite this article**: Lin, Y.-T. *et al*. Association between acetylcholinesterase inhibitors and risk of stroke in patients with dementia. *Sci. Rep.* **6**, 29266; doi: 10.1038/srep29266 (2016).

## Supplementary Material

Supplementary Information

## Figures and Tables

**Table 1:** Propensity score-matched baseline characteristics among dementia patients without ischemic stroke receiving acetylcholinesterase inhibitors or not.

	Patients using AChEIs (n = 5182)		Patients not using AChEIs (n = 5182)		*P*-value
	N	%	n	%	
Age, years					>0.999
50–59	5	0.1	5	0.1	
60–69	219	4.2	219	4.2	
70–79	877	16.9	877	16.9	
≥80	4081	78.8	4081	78.8	
Sex					>0.999
Men	2221	42.9	2221	42.9	
Women	2961	57.1	2961	57.1	
Urbanization level					0.198
City area	3771	72.8	3829	73.9	
Rural area	1411	27.2	1353	26.1	
Socioeconomic status					0.046
Low	2810	54.2	2700	52.1	
Moderate	1141	22	1237	23.9	
High	1231	23.8	1245	24	
Comorbidities					
Diabetes mellitus	1586	30.6	1506	29.1	0.086
Hypertension	3033	58.5	2864	55.3	<0.001
Hyperlipidemia	1125	21.7	1072	20.7	0.203
Coronary artery disease	1769	34.1	1682	32.5	0.070
Heart failure	802	15.5	766	14.8	0.324
Atrial fibrillation	189	3.6	176	3.4	0.489
Peripheral artery disease	199	3.8	180	3.5	0.320
COPD	1908	36.8	1851	35.7	0.244
Chronic kidney disease	308	5.9	301	5.8	0.770
Malignancy	455	8.8	448	8.6	0.807
Depression	940	18.1	843	16.3	0.012
AChEIs, cDDD					
<28 cDDDs	286	5.5			
28–365 cDDDs	2424	46.8			
≥365 cDDDs	2472	47.7			

Abbreviations: COPD, chronic obstructive pulmonary disease; AChEIs, cholinesterase inhibitors; SD, standard deviation; cDDD, cumulative defined daily dose.

**Table 2:** Follow-up duration, numbers, and incidence rate of ischemic stroke among dementia patients using and not using acetylcholinesterase inhibitors.

Clinical outcome	Patients using AChEIs (n = 5182)	Patients not using AChEIs (n = 5182)
Total follow-up person-years	26,077.03	26,122.93
Mean follow-up time (y)	5.03	5.04
No. of ischemic stroke	418	629
Incidence rate per 10,000 person-years (95% CI)	160.3	240.8
	(145.5–176.2)	(222.5–260.2)

CI, confidence interval; AChEIs, acetylcholinesterase inhibitors.

**Table 3:** Hazard ratio for ischemic stroke among acetylcholinesterase inhibitors users and nonusers in dementia cohort.

Clinical outcome	Hazard ratio	95% Confidence interval
Ischemic stroke*		
Model 1	0.655	0.579–0.742
Model 2	0.508	0.434–0.594
Death		
Model 1	0.947	0.875–1.024
Model 2	0.996	0.872–1.137

Model 1: Propensity score–matched Cox proportional hazards model. Model 2: Propensity score–matched Cox proportional hazards model and further adjustme nt for medications in observation period.

^*^Adjusted for competing death risk.

**Table 4:** Incidence rate, crude and adjusted HRs of ischemic stroke associated with acetylcholinesterase inhibitors use during the follow-up period in the propensity score–matched dementia cohort.

	No. of patients with ischemic stroke	Incidence rate (95% CI)	Crude		Adjusted*		*P* for trend
			HR (95% CI)	*P*-value	HR (95% CI)	*P*-value	
Total AChEIs use duration							<0.001
Nonuser (<28 cDDDs)	646	235.4 (217.7–254.2)	Reference		Reference		
User (28–365 cDDDs)	227	195.9 (171.7–222.7)	0.757 (0.621–0.921)	0.006	0.646 (0.567–0.736)	<0.001	
User (>365 cDDDs)	174	132.1 (113.6–152.9)	0.517 (0.416–0.641)	<0.001	0.587 (0.512–0.672)	<0.001	

Abbreviations: AChEIs, acetylcholinesterase inhibitors.

^*^Propensity score–matched time-dependent Cox proportional hazards model and further adjustment for medications in observation period.
