# Supplementary material for: Association between acetylcholinesterase inhibitors and risk of stroke in patients with dementia
Source: Sci Rep. 2016 Jul 5;6:29266. doi: 10.1038/srep29266 (PMC4932523; doi:10.1038/srep29266)
Supplement: Supplementary Information [file srep29266-s1.pdf]

Association between acetylcholinesterase inhibitors and risk of stroke in patients with dementia

Yi-Ting Lin[1,5,6], Ping-Hsun Wu[1,7], Cheng-Sheng Chen[3,8], Yi-Hsin Yang[2], Yuan-Han Yang[4,9,10]

[1]Graduate Institute of Clinical Medicine, College of Medicine, Kaohsiung Medical University, Kaohsiung, Taiwan

[2]School of Pharmacy, College of Pharmacy, Kaohsiung Medical University, Kaohsiung, Taiwan

[3]Department of Psychiatry, Kaohsiung Medical University, Kaohsiung, Taiwan

[4]Department of Neurology, Kaohsiung Medical University, Kaohsiung, Taiwan

[5]Department of Family Medicine, Kaohsiung Municipal Hsiao–Kang Hospital, Kaohsiung

[6]Department of Family Medicine, Kaohsiung Municipal Hsiao–Kang Hospital, Kaohsiung

[7]Division of Nephrology, Department of Internal Medicine, Kaohsiung Medical University Hospital, Kaohsiung

[8]Department of Psychiatry, Kaohsiung Medical University Hospital, Kaohsiung, Taiwan

[9]Department of Neurology, Kaohsiung Medical University Hospital, Kaohsiung,

Taiwan

[10]Department of Neurology, Kaohsiung Municipal Ta-Tung Hospital, Kaohsiung, Taiwan

Corresponding author:

Yuan-Han Yang MD, PhD

Department of Neurology, Kaohsiung Medical University Hospital

No.100,Tzyou 1st Road Kaohsiung 807 , Kaohsiung, Taiwan

Telephone number: +886-7-3121101 ext 5408

Fax number: +886-7-3162158

E-mail address: endless@kmu.edu.tw

Supplementary Online Content

Supplemental Figure S1. Study flow chart

Supplemental Figure S2. Cumulative incidences of ischemic stroke of Alzheimer's disease patients who were or were not treated with acetylcholinesterase inhibitors. Data were compiled after adjustment for competing mortality. For cumulative incidences of ischemic stroke, calculation and comparison in competing risk data ratios were conducted using modified Kaplan-Meier and Gray methods.

Supplementary Table S1. ICD-9-CM codes used to identify clinical conditions

Supplementary Table S2. Drug (Anatomical Therapeutic Chemical code) concomitant prescriptions in the present study

Supplementary Table S3. Baseline characteristics of Alzheimer's disease patients receiving acetylcholinesterase inhibitors or not matched by age and sex

Supplementary Table S4. Follow-up duration, numbers, and incidence rate of ischemic stroke among Alzheimer's disease patients using and not using acetylcholinesterase inhibitors

Supplementary Table S5. Hazard ratio for ischemic stroke among acetylcholinesterase inhibitors users and nonusers in Alzheimer's disease cohort using various analytical models

Supplementary Table S6. Incidence rate, crude and adjusted HRs of ischemic stroke associated with acetylcholinesterase inhibitors use during the follow-up period in the age- and sex-matched Alzheimer's disease cohort

Supplemental Figure S1. Study flow chart

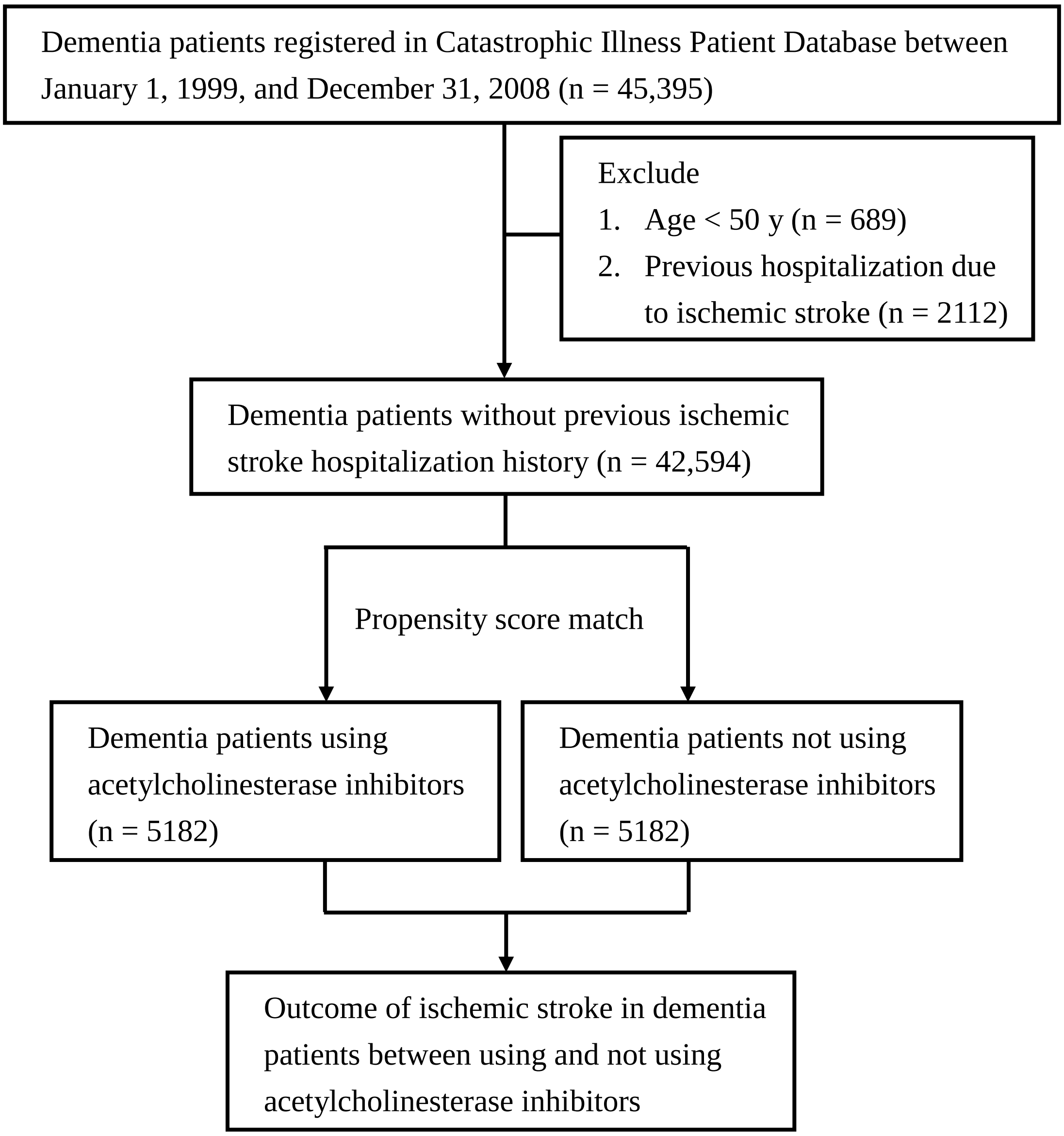

Supplemental Figure S2. Cumulative incidences of ischemic stroke of Alzheimer's disease patients who were or were not treated with acetylcholinesterase inhibitors. Data were compiled after adjustment for competing mortality. For cumulative incidences of ischemic stroke, calculation and comparison in competing risk data ratios were conducted using modified Kaplan-Meier and Gray methods.

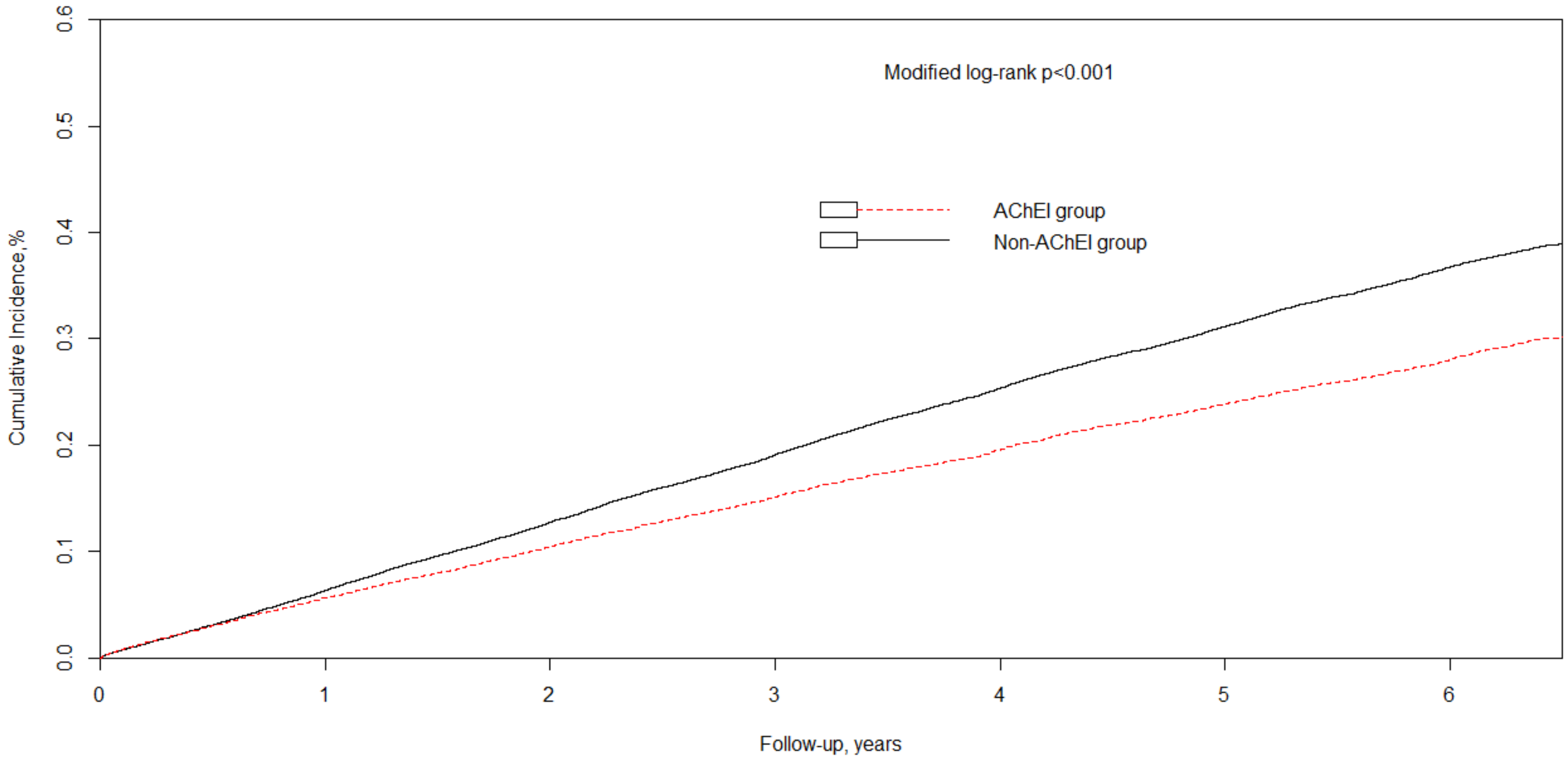

Supplementary Table S1. ICD-9-CM codes used to identify clinical conditions

Diagnosis	Corresponding ICD-9 codes
Dementia	290, 331.0
Ischemic stroke	433, 434, 436
Diabetes mellitus	250
Hypertension	401–405
Hyperlipidemia	272
Coronary artery disease	410–414, 429.2
Heart failure	398.91, 402.01, 402.11, 402.91, 404.01, 404.03, 404.11, 404.13, 404.91, 404.93, 428
Atrial fibrillation	427.3
Peripheral artery disease	440.2–440.4, 443.9
Chronic obstructive pulmonary disease	491, 492, 496
Chronic kidney disease	250.4, 274.1, 403.1, 404.2, 404.3, 580, 582, 583, 585, 586, 587
Malignancy	140–208
Depression	296.2, 296.3, 296.82, 300.4, 309.0, 309.1, 311

ICD, international classification of disease

Supplementary Table S2. Drug (Anatomical Therapeutic Chemical code) concomitant prescriptions in the present study

Drug type	ATC classification system codes	Drug name
Antiplatelets	B01AC04, B01AC05, B01AC06	Clopidogrel, Ticlopidine, Acetylsalicylic acid (Aspirin)
Dipyridamole	B01AC07	Dipyridamole
Anticoagulants	B01AA03	Warfarin
Angiotensin-converting enzyme inhibitors (ACEIs)	C09A, C09B	Captopril, Enalapril, Lisinopril, Perindopril, Ramipril, Quinapril, Benazepril, Cilazapril, Fosinopril, Imidapril
Angiotensin receptor blockers (ARBs)	C09C, C09D	Candesartan, Irbesartan, Losartan, Olmesartan, Telmisartan, Valsartan
Beta-blockers	C07A	Labetalol, Pindolol, Acebutolol, Alprenolol, Atenolol, Betaxolol, Bisoprolol, Carteolol, Carvedilol, Nadolol, Metoprolol, Oxprenolol, Propranolol, Sotalol, Timolol, Metipranolol, Esmolol
Thiazides	C03AA03, C03AA06	Hydrochlorothiazide, Trichlormethiazide
Calcium channel blockers (CCBs)	C08C, C08D, C08E	Nifedipine, Nicardipine, Felodipine, Amlopidine, Isradipine, Diltiazem, Verapamil
Statins	C10AA	Atorvastatin, Fluvastatin, Lovastatin, Pravastatin, Rosuvastatin, Simvastatin
Fibrates	C10AB	Bezafibrate, Clofibrate, Etofibrate, Gemfibrozil, Fenofibrate, Simfibrate
Traditional nonsteroidal anti-inflammatory drugs (traditional NSAIDs)	M01AA, M01AB, M01AC, M01AE, M01AG, M01AX	Acemetacin, Aloclofenac, Alminoprofen, Benzydamine, Diclofenac, Diflunisal, Etodolac, Etofenamate, Fenbufen, Flufenamate, Flufenamic acid, Flurbiprofen, Ibuprofen, Iclofenac, Indomethacin, Ketoprofen, Ketorolac, Meclofenamate, Meclofenamic acid, Naproxen, Piroxicam, Spirin, Sulindac, Niflumic acid, Tenoxicam, Tiaprofenic acid, Tiaramide, Tolmetin, Tolfenamic acid, Mepirizole
Cyclooxygenase-2- selective inhibitors (COX-2 inhibitors)	M01AH	Celecoxib, Meloxicam, Rofecoxib, Etoricoxib, Nimesulide
Proton pump inhibitors (PPIs)	A02BC	Omeprazole, Esomeprazole, Pantoprazole, Lansoprazole, Rabeprazole
H-2 receptor antagonists	A02BA	Cimetidine, Ranitidine, Famotidine
Antidepressants	N06A	Fluoxetine, Citalopram, Paroxetine, Sertraline, Fluvoxamine, Escitalopram, Imipramine, Clomipramine, Amitriptyline, Doxepin, Dosulepin, Maprotiline, Melitracen, Nortriptyline, Moclobemide, Duloxetine, Milnacipran, Venlafaxine, Trazodone, Mirtazapine, Bupropion

Antipsychotics	N05A	Chlorpromazine, clopenthixol, clothiapine, flupenthixol, fluphenazine, haloperidol, levomepromazine, loxapine, methotrimeprazine, perphenazine, pimozide, pipotiazine, prochlorperazine, sulpiride, trifluoperazine, thioridazine, amisulpride, aripiprazole, clozapine, olanzapine, quetiapine, risperidone, ziprasidone, and zotepine

Supplementary Table S3. Baseline characteristics of Alzheimer's disease patients receiving acetylcholinesterase inhibitors or not matched by age and sex

	AD Patients using AChEIs (n = 2649)		AD Patients not using AChEIs (n = 2649)		*P*-value
	N	%	n	%	
**Age, years**					0.984
50–59	80	3	75	2.8	
60–69	394	14.9	394	14.9	
70–79	1222	46.1	1222	46.2	
≥80	952	36	952	36	
**Sex**					> 0.999
Men	1101	41.6	1101	41.6	
Women	1548	58.4	1548	58.4	
**Urbanization level**					0.046
City area	2049	77.3	1987	75	
Rural area	600	22.7	662	25	
**Socioeconomic status**					0.074
Low	1444	54.5	1525	57.6	
Moderate	605	22.8	574	21.7	
High	600	22.7	550	20.8	
**Comorbidities**					
Diabetes mellitus	772	29.1	206	7.8	<0.001
Hypertension	1487	56.1	417	15.7	<0.001
Hyperlipidemia	816	30.8	177	6.7	<0.001
Coronary artery disease	938	35.4	242	9.1	<0.001
Heart failure	338	12.8	99	3.7	<0.001
Atrial fibrillation	78	2.9	26	1	<0.001
Peripheral artery disease	123	4.6	22	0.8	<0.001
COPD	918	34.7	279	10.5	<0.001
Chronic kidney disease	144	5.4	48	1.8	<0.001
Malignancy	317	12	85	3.2	<0.001
Depression	630	23.8	147	5.5	<0.001
**Concomitant medications**					
Antiplatelets	1029	38.8	272	10.3	<0.001
Dipyridamole	412	15.51	115	4.33	<0.001
Warfarin	61	2.30	17	0.64	<0.001
ACEIs	578	21.8	164	6.2	<0.001
ARBs	565	21.3	146	5.5	<0.001
Beta-blockers	818	30.9	202	7.6	<0.001

	AD Patients using AChEIs (n = 2649)		AD Patients not using AChEIs (n = 2649)		*P*-value
	N	%	n	%	
Thiazides	660	24.9	182	6.9	<0.001
CCBs	1148	43.3	307	11.6	<0.001
Statins	910	34.4	195	7.4	<0.001
Fibrates	110	4.2	35	1.3	<0.001
Traditional NSAIDs	1101	41.6	290	10.9	<0.001
COX-2 inhibitors	713	26.9	168	6.3	<0.001
PPIs	360	13.6	95	3.6	<0.001
H-2 receptor antagonists	190	7.2	49	1.8	<0.001
Antidepressants	781	29.5	173	6.5	<0.001
Antipsychotics					
**AChEIs, cDDD**					
<28 cDDDs	113	4.3			
28–365 cDDDs	1124	42.4			
≥365 cDDDs	1412	53.3			

Abbreviations: AD, Alzheimer's disease; COPD, chronic obstructive pulmonary disease; ACEIs, angiotensin-converting-enzyme inhibitors; ARBs, angiotensin receptor blockers; CCBs, calcium-channel blockers; PPIs, proton-pump inhibitors; H2- receptor antagonists, histamine-2 receptor antagonists; Traditional NSAIDs, traditional nonsteroidal anti-inflammatory drugs; COX-2 inhibitors, cyclooxygenase-2-selective nonsteroidal anti-inflammatory inhibitors; AChEIs, acetylcholinesterase inhibitors

Supplementary Table S4. Follow-up duration, numbers, and incidence rate of ischemic stroke among Alzheimer's disease patients using and not using acetylcholinesterase inhibitors

Clinical outcome	AD Patients using AChEIs (n = 2649)	AD Patients not using AChEIs (n = 2649)
Total follow-up, person-years	13,700	13,753
No. of ischemic stroke	228	266
Incidence rate per 10,000 person-years (95% CI)	166.4 (145.8–189.1)	193.1 (171.2–217.7)

Abbreviations: CI, confidence interval; AD, Alzheimer's disease

Supplementary Table S5. Hazard ratio for ischemic stroke among acetylcholinesterase inhibitors users and nonusers in Alzheimer's disease cohort using various analytical models

Clinical outcome	Adjusted hazard ratio	95% Confidence interval
Ischemic stroke*		
Model 1	0.626	0.512–0.767
Model 2	0.615	0.498–0.760
Death		
Model 1	0.927	0.829–1.037
Model 2	0.897	0.801–1.004

*Adjusted for competing death risk

Model 1: adjustment for urbanization, socioeconomic status, and comorbidities

Model 2: adjustment for Model 1 and concomitant medications

Supplementary Table S6. Incidence rate, crude and adjusted HRs of ischemic stroke associated with acetylcholinesterase inhibitors use during the follow-up period in the age- and sex-matched Alzheimer's disease cohort

			Crude		Adjusted*		
	No. of patients	Incidence rate (95% CI)	HR (95% CI)	*P*-value	HR (95% CI)	*P*-value	*P* for Trend
**Ischemic stroke**							
Total AChEI use duration							
Nonuser (<28 cDDD)	269	189.2 (167.6–212.9)	Reference		Reference		<0.001
User (28–365 cDDDs)	112	203.3 (168.2–243.7)	0.918 (0.675–1.248)	0.584	0.779 (0.610–0.994)	0.045	
User (>365 cDDDs)	113	146.2 (121.0–175.1)	0.714 (0.541–0.944)	0.018	0.585 (0.456–0.750)	<0.001	

Abbreviations: AChEIs, acetylcholinesterase inhibitors

*Adjustment for urbanization, socioeconomic status, comorbidities, and concomitant medications
